# Massively parallel single-cell genomics of microbiomes in rice paddies

**DOI:** 10.3389/fmicb.2022.1024640

**Published:** 2022-11-03

**Authors:** Wataru Aoki, Masato Kogawa, Shuhei Matsuda, Keisuke Matsubara, Shintaro Hirata, Yohei Nishikawa, Masahito Hosokawa, Haruko Takeyama, Toru Matoh, Mitsuyoshi Ueda

**Affiliations:** ^1^Division of Applied Life Sciences, Graduate School of Agriculture, Kyoto University, Kyoto, Japan; ^2^Research Organization for Nano and Life Innovation, Waseda University, Tokyo, Japan; ^3^WORLD INTEC CO., LTD., Fukuoka, Japan; ^4^Kyoto Agriculture Research Institute KARI, Kyoto, Japan; ^5^Computational Bio Big-Data Open Innovation Laboratory, National Institute of Advanced Industrial Science and Technology, Tokyo, Japan; ^6^Department of Life Science and Medical Bioscience, Waseda University, Tokyo, Japan; ^7^Institute for Advanced Research of Biosystem Dynamics, Waseda Research Institute for Science and Engineering, Tokyo, Japan

**Keywords:** massively parallel single-cell sequencing, microbiomes, rice paddies, bacterial community compositions, comparative genomics

## Abstract

Plant growth-promoting microbes (PGPMs) have attracted increasing attention because they may be useful in increasing crop yield in a low-input and sustainable manner to ensure food security. Previous studies have attempted to understand the principles underlying the rhizosphere ecology and interactions between plants and PGPMs using ribosomal RNA sequencing, metagenomic sequencing, and genome-resolved metagenomics; however, these approaches do not provide comprehensive genomic information for individual species and do not facilitate detailed analyses of plant–microbe interactions. In the present study, we developed a pipeline to analyze the genomic diversity of the rice rhizosphere microbiome at single-cell resolution. We isolated microbial cells from paddy soil and determined their genomic sequences by using massively parallel whole-genome amplification in microfluidic-generated gel capsules. We successfully obtained 3,237 single-amplified genomes in a single experiment, and these genomic sequences provided insights into microbial functions in the paddy ecosystem. Our approach offers a promising platform for gaining novel insights into the roles of microbes in the rice rhizomicrobiome and to develop microbial technologies for improved and sustainable rice production.

## Introduction

Considering the global population growth and climate change, there is an urgent need to address food security by increasing crop yield in a low-input and sustainable manner. Recently, the use of beneficial plant-associated microbes in agriculture has attracted increasing attention (Backer et al., 2018). Rhizosphere microbes play important roles in the ecological fitness of plants, and beneficial plant growth-promoting microbes (PGPMs) enhance plant growth and health (Franche et al., 2009; Lugtenberg and Kamilova, 2009; Berendsen et al., 2012; Kumar and Dubey, 2020). For example, some PGPMs promote plant growth by providing ammonia *via* nitrogen fixation (Herridge et al., 2008); improving the uptake of minerals such as phosphorus (van der Heijden et al., 1998; Lavakush et al., 2014), iron (Sharma et al., 2013), and zinc (Shakeel et al., 2015); and producing growth regulators such as plant hormones (Lugtenberg and Kamilova, 2009). PGPMs also confer tolerance to plants against abiotic stresses (Meena et al., 2017; Tiwari et al., 2017) such as drought (Ruíz-Sánchez et al., 2011; Shukla et al., 2012; Pandey et al., 2016), heavy metals (Mishra et al., 2017; Tiwari and Lata, 2018), and salinity (Jogawat et al., 2016; Qin et al., 2016; Yuan et al., 2016). Furthermore, several PGPMs induce systemic resistance against biotic stresses, priming plant defense against various pathogens and insect herbivores (Pieterse et al., 2014; Mhlongo et al., 2018; Syed Ab Rahman et al., 2018). PGPMs represent low-cost agricultural inputs, which reduce the use of synthetic fertilizers and agrochemicals; therefore, PGPM-based approaches may be instrumental in realizing economic and sustainable agriculture.

Rice is one of the most important staple foods worldwide and has high calorific value (Elert, 2014); therefore, developing PGPM-based biostimulants for sustainable paddy ecosystems will aid in addressing food security (Yanni et al., 1997; Chi et al., 2005; Ding et al., 2019; Kim and Lee, 2020). To this aim, researchers have attempted to understand the principles underlying the rhizosphere ecology of rice. High-throughput sequencing of 16S, 18S, and internal transcribed spacer regions of ribosomal RNA (rRNA) genes revealed that microbial diversity and its dynamics in rice rhizosphere are affected by various factors (Edwards et al., 2015, 2018) such as geographical location (Chen et al., 2017), soil type (Xu et al., 2020), nutrient status (Ikeda et al., 2014; Chen et al., 2017; Wang et al., 2017; Dong et al., 2021), rice genotype (Shenton et al., 2016; Zhang et al., 2019), growth stage (Breidenbach et al., 2015; Imchen et al., 2019), and other factors (Jiang et al., 2016; Santos-Medellín et al., 2017). Furthermore, metagenomic sequencing was used to characterize the functional interactions between rice plants and the rhizomicrobiome at the molecular level (Okubo et al., 2014; Zheng et al., 2014; Bhattacharyya et al., 2016; Sengupta et al., 2017; Wang et al., 2018; Zhong et al., 2020). However, metagenomic sequencing does not provide genomic information for individual species and therefore does not allow detailed analyses of plant–microbe interactions. To address this problem, some studies obtained the genomic sequences of microbial isolates (Krause et al., 2006; Midha et al., 2015; Chaudhry et al., 2016; Hwangbo et al., 2016; Lin et al., 2016; Battu and Ulaganathan, 2020) or used genome-resolved metagenomics (Erkel et al., 2006; Xu et al., 2021), enabling a deeper examination of plant–microbe interactions. However, these approaches have certain limitations. Genomic sequencing of microbial isolates is not applicable to unculturable microorganisms, and genome-resolved metagenomics often suffers from binning errors of metagenomic reads (Mallawaarachchi et al., 2021). Therefore, it is necessary to develop a highly precise, scalable, and universal approach to obtain the genomic information of individual microbial species in rice rhizosphere.

Recently, high-throughput single-cell genomic sequencing has opened up new opportunities to understand the ecology of microbiomes. In contrast to the metagenome-assembled genome (MAG) generation, single-cell genomics does not require microbial population clonality but instead retrieves individual microbial genome sequence as single-amplified genome (SAG) from a complex microbial community. SAG is not a population-common sequence like MAG, but provides a strain-resolution genome derived from a single microorganism. We have developed a massively parallel single microbial genome sequencing technique, called SAG-gel, to obtain SAGs without microbial cultivation or a metagenomic binning approach (Chijiiwa et al., 2020; Arikawa et al., 2021; Hosokawa et al., 2022; Ide et al., 2022; Nishikawa et al., 2022). The SAG-gel platform uses microfluidic-generated gel capsules that allow single-cell encapsulation and single-cell genome amplification in a gel capsule. We have demonstrated its applicability for obtaining soil microbial genomes (Yoda et al., 2020; Nishikawa et al., 2022).

In the present study, we developed a pipeline to analyze the genomic diversity of the rice rhizosphere microbiome at single-cell resolution. We isolated microbial cells from paddy soil and determined their genomic sequences by using massively parallel whole-genome amplification in microfluidic-generated gel capsules. We successfully obtained 3,237 SAGs in a single experiment. The genomic sequences provided novel insights into the functions of microbes in the paddy ecosystem. Our approach offers a promising platform for gaining insights into the functions of microbes in the rice rhizomicrobome and to develop microbial technologies for improved and sustainable rice production.

## Results and discussion

### Cultivation of rice

**Figure 1:**
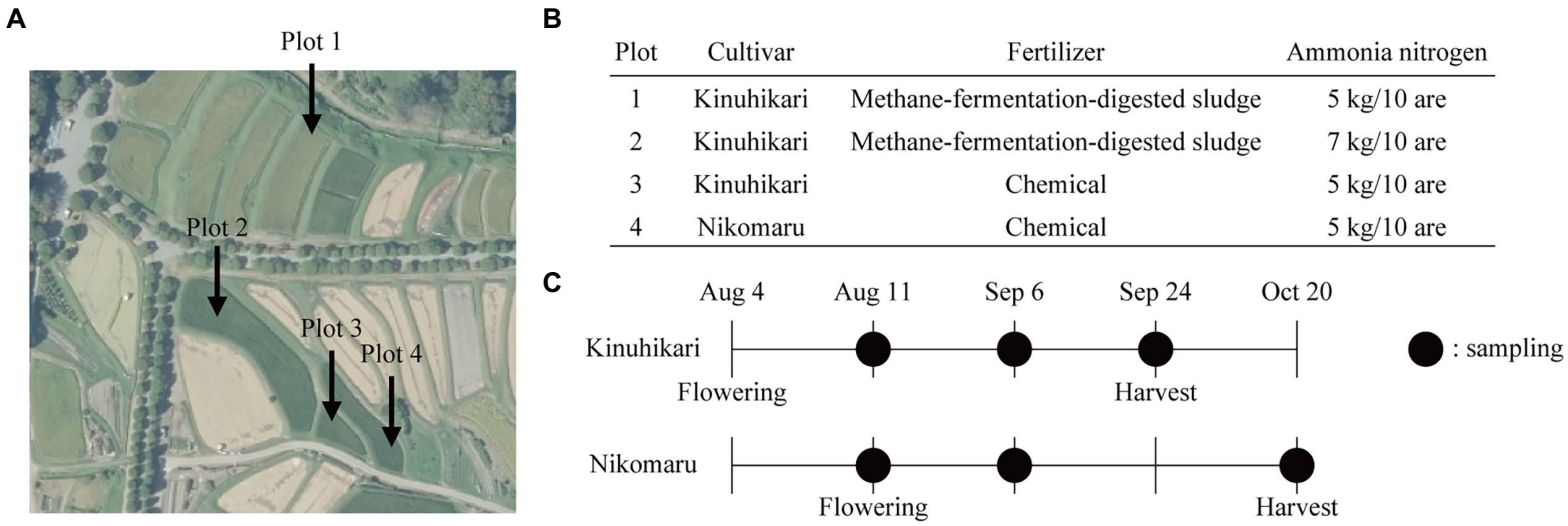
Field experiment design and sample collection. **(A)** The aerial photograph of the rice paddies used in this study. The photograph was taken by the Geospatial Information Authority of Japan (https://maps.gsi.go.jp/vector/#17/35.052689/135.801694/&ls=photo&disp=1&d=l). **(B)** Design of the field experiment. Each plot differs in types of cultivars, fertilizers, or the amounts of ammonia nitrogen. **(C)** Sampling time points of bulk and rhizosphere soils. Black-filled circles indicate sampling time points.

In this study, we aimed to develop a highly precise, scalable, and universal approach to obtain genomic information of individual species in the rice rhizosphere. As a model, we chose four plots of rice paddies (plots 1–4) in Shugakuin Imperial Villa in Kyoto city (Figure 1A). The rice paddies were composed of loamy soils and had similar soil chemical properties (Supplementary Table S1). The four plots differed in cultivar type, fertilizer type, or quantity of ammonia nitrogen (Figure 1B). We collected soil samples (bulk or rhizosphere) at three sampling time points to assess the effects of growth stage on the differences in bacterial communities (Figure 1C). Grain yield in the four plots is summarized in Table 1.

**Table 1:** Grain yield of each plot.

Plot	Area (m^2^)	Paddy yield (kg)	Paddy yield/area (g/m^2^)	Brown rice yield/area (g/m^2^)
1	417	228	547	416
2	700	428	611	446
3	126	54	429	326
4	246	134	545	420

### Structure of microbial communities

**Figure 2:**
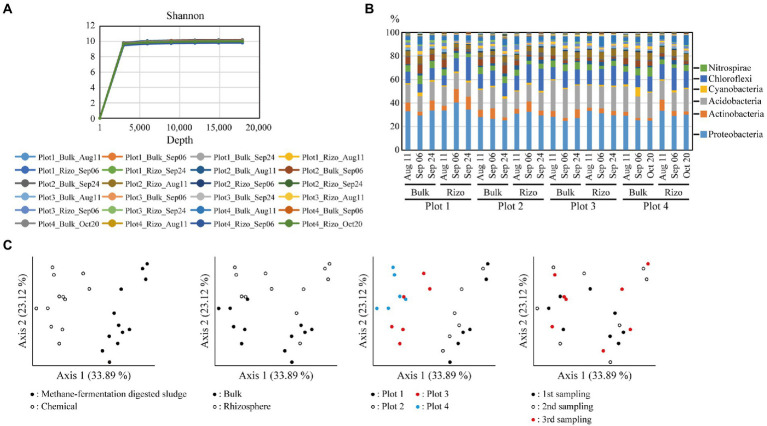
Diversity of soil bacterial communities. **(A)** Rarefaction curves of Shannon index on the sequencing depth based on the alpha diversity analysis. **(B)** Bacterial community compositions. Relative abundance of each phylum is shown in the graph, and the six major phyla are described to the right. **(C)** Weighted Unifrac principal coordinate analysis (PCoA) of the bacterial communities. Each graph is annotated according to each variable (cultivar., soil fraction, fertilizer type, or sampling time point). Rizo indicates rhizosphere.

Bulk and rhizosphere soil samples were collected at each sampling time point, and their 16S rRNA sequences were analyzed. A rarefaction curve of Shannon index reached a plateau, indicating that the sequencing depth in this study was sufficient to illustrate the bacterial diversity in rice paddies (Figure 2A). The bacterial community composition at the phylum level showed that Proteobacteria was the most abundant (Figure 2B); this observation is consistent with previous findings (Edwards et al., 2015; Xu et al., 2020). Differences in microbial communities were evaluated using weighted UniFrac principal coordinate analysis (PCoA) at the amplicon sequence variant (ASV) level (Figure 2C). Samples from plots with different fertilizers (methane-fermentation–digested sludge or chemical fertilizer) occupied distinct areas along axis 1 of the PCoA plot, indicating that, of the four variables (cultivar., soil fraction, fertilizer, or sampling time point), fertilizer type had the most significant impact on microbial communities. Samples from different soil fractions (bulk or rhizosphere) occupied distinct areas along axis 2, indicating that soil fraction type had a significant impact on microbial communities. Plot 3 (Kinuhikari) and plot 4 (Nikomaru) samples occupied similar areas of the PCoA graph, indicating that cultivar type had a lower impact on microbial communities. Samples from various time points were randomly distributed on the PCoA plot, suggesting that sampling time did not strongly affect microbial communities. Our findings are supported by previous studies, which showed that fertilizer type (inorganic or organic) significantly changed soil bacterial community diversity (Wang et al., 2017) and that soil environment type had a greater effect on microbial communities than sampling time points (growth stages; Breidenbach et al., 2015).

### Single-cell genome analysis of paddy soil microbes

**Figure 3:**
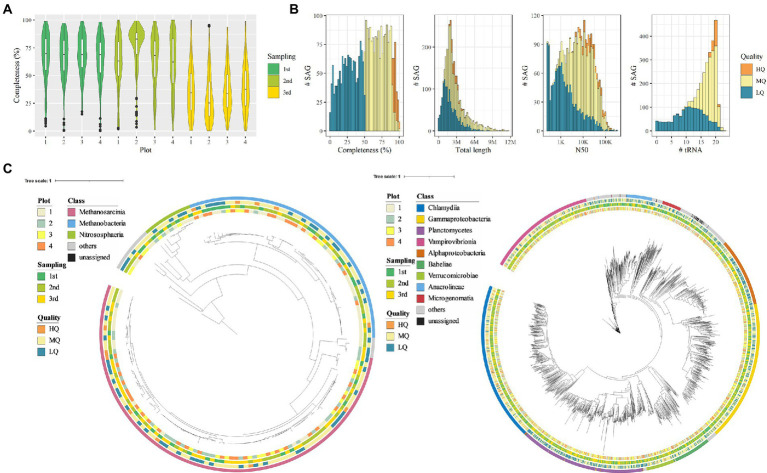
Quality and taxonomy classification of bacterial and archaeal single-amplified genomes (SAGs). **(A)** SAG completeness per sampling point. **(B)** Histogram of genome completeness, total length, N50, and the number of tRNAs corresponding to bacterial and archaeal SAGs. **(C)** Taxonomic tree based on archaeal/bacterial single-copy marker genes of SAGs (left, archaea; right, bacteria). The external color bars show the metadata and taxonomical annotation result (from inwards to outwards: plot, sampling time point, genome quality, and class-level annotation).

Single-amplified genomes (SAGs) of soil microbes were obtained from 12 soil samples (four plots × three sampling time points) that correspond to the 16S rRNA amplicon sequencing. A total of 4,608 SAGs (868 Gb) were obtained, and 3,237 SAGs were classified as high-quality (HQ, 181 SAGs), medium-quality (MQ, 1,694 SAGs), or low-quality genomes (LQ, 1,362 SAGs; Figure 3A). The remaining SAG data were either contaminated or had no detectable microbial marker genes. The average total length of the HQ, MQ, and LQ genomes was 2.94, 3.11, and 1.92 Mb, respectively, and the average N50 was 31.1, 18.7, and 5.7 kb, respectively (Figure 3B). On average, 19.6, 17.6, and 10.8 tRNAs were detected in the HQ, MQ, and LQ genomes, respectively. In addition, 16S rRNA gene sequences were detected in 68% of the SAGs (2,200 SAGs), demonstrating the advantage of SAG analysis in that it is easy to link whole genome information to 16S rRNA gene sequences. A comparison of the quality of the acquired SAG data for each soil sample showed that the SAG sets obtained from soil samples acquired during the first and second sampling events showed 66.7% and 64.1% completeness on average, respectively. In contrast, the SAG set obtained from soil samples acquired during the third sampling event averaged 34.5% completeness, which was lower than that observed in the first two events (Figure 3A). We attributed the lower quality in this third data set to the fact that the third sampling was performed after draining the paddy soil, which might increase the percentage of dead cells in the sample. The SAG data acquired in this study, even for the LQ SAGs, had an average total length of 1.92 Mb, suggesting that relatively higher quality SAG data were acquired compared with the soil microbial SAG data obtained in previous studies (Rinke et al., 2014; Stepanauskas et al., 2017; Nishikawa et al., 2022).

### Taxonomic profiling of SAGs

As a result of phylogenetic annotation based on the Genome Taxonomy Database (GTDB), the soil microbial SAGs were classified into 2,616 bacterial SAGs, 164 archaeal SAGs, and others into the unknown at domain-level phylogenetic annotation (Figure 3C). Corresponding to the results of the 16S rRNA gene sequencing, Proteobacteria SAGs were the most abundant (*n* = 664). On the other hand, we identified a lower number of SAGs corresponding to other phyla such as Acidobacteria and Chloroflexi that were highly abundant in the 16S rRNA gene sequencing (68 and 105 SAGs, respectively). The differences in phylogenetic profiles determined from 16S rRNA gene sequencing and SAGs may be due to the differences in sample processing, such as freezing of soil samples used in SAG acquisition or enzymatic lysis. In addition, the archaeal SAGs were mainly from methanogenic archaea such as Methanobacteria, Methanomicrobia, and Methanosarcinia, which are known to cause methanogenesis in rice paddies (Lee et al., 2014; Alpana et al., 2017; Asakawa, 2021). SAGs corresponding to the families Methanotrichaceae and Methanobacteriaceae (*n* = 85 and 52, respectively) accounted for most archaeal SAGs.

### Comparative genomics of the methanogenic archaeal communities

**Figure 4:**
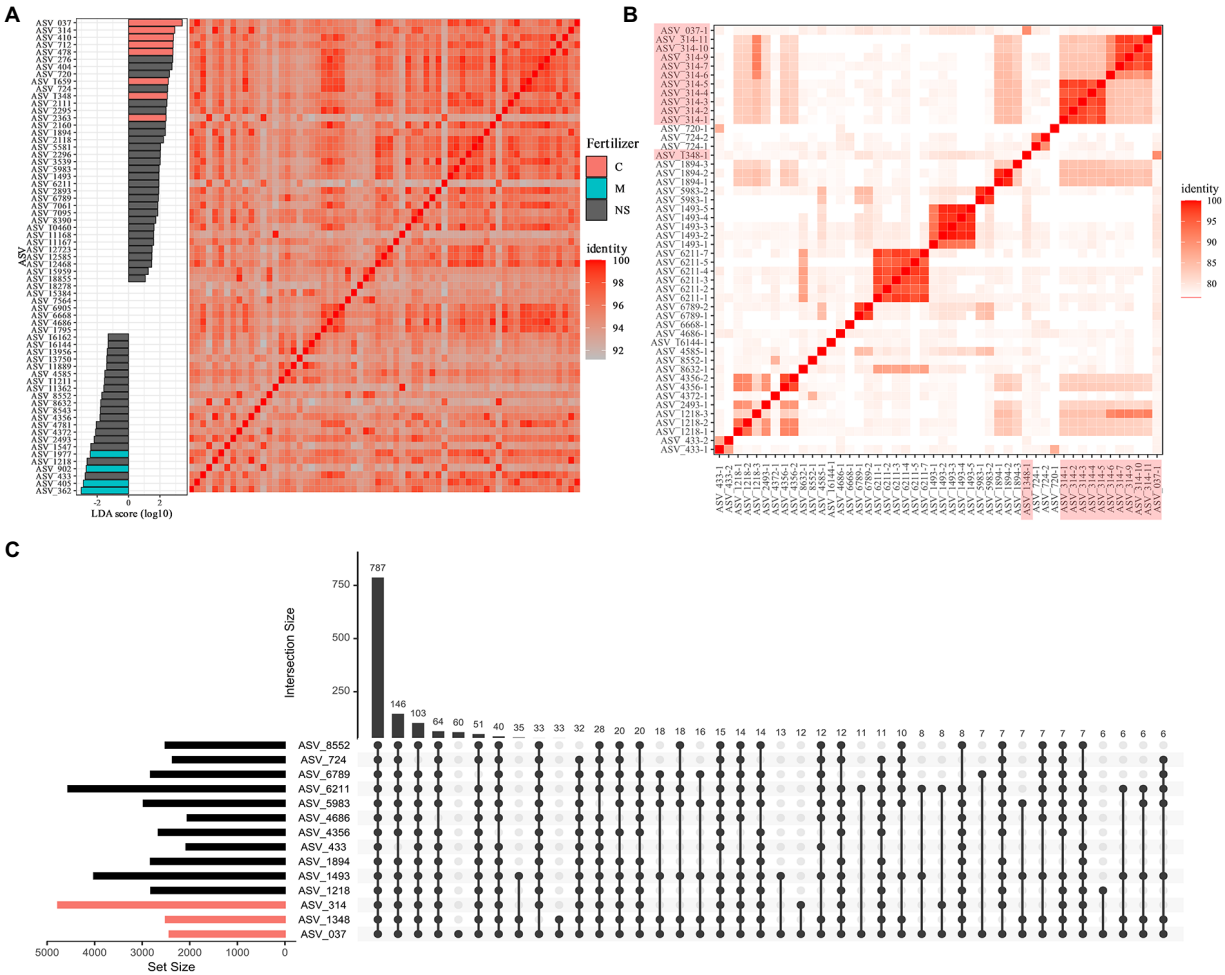
Comparative genomics of methanogenic archaea belonging to the genus Methanothrix. **(A)** Linear discriminant analysis effect size of Methanothrix amplicon sequence variants (ASVs). Red and blue bars represent ASVs that are significantly more abundant in chemically fertilized paddies and organically fertilized paddies, respectively. The heat map shows the homology among ASVs representative of 16S rRNA gene partial sequences. **(B)** Average nucleotide identity (ANI) heat map of high-quality (HQ) and medium-quality (MQ) SAGs with 16S rRNA gene sequences corresponding to Methanothrix ASVs. Axis labels highlighted in red indicate SAGs corresponding to chemical fertilizer-specific ASVs. **(C)** Upset diagram based on ortholog analysis between SAG datasets corresponding to each metanogenic archeal ASV. Red bars indicate SAGs corresponding to ASVs specific to chemically fertilized paddies.

The methanogenic archaeal communities contribute significantly to methanogenesis in rice paddies, and these communities exhibit co-occurrence patterns across different rice paddy locations (Li et al., 2021). Hence, we focused on analyze ASVs derived from the methanogenic archaeal communities. The key ASVs considered to originate from methanogenic archaea were detected in all paddy fields. In contrast, certain methanogenic archaeal ASVs showed differential abundance among paddy fields. For example, in rice paddies with different fertilizer applications, where a clear difference was observed in PCoA, eight and four ASVs corresponding to the genus Methanothrix were found to be significantly more abundant in chemically fertilized and organically fertilized paddies, respectively (Figure 4A). The methane-fermentation-digested sludge contains different chemical species compared with the chemical fertilizer, which may affect the resultant microbial communities. Comparison of ASVs showed no relationship between sequence similarity and fertilizer application conditions. SAGs with 16S rRNA gene sequences showing ≥97% homology to Methanothrix ASVs were collected from the archaeal SAG; 50 Methanothrix SAGs were identified, including 13 Methanothrix SAGs corresponding to three ASVs (037, 314, 1,348) common in chemically fertilized paddy fields.

The average nucleotide identities (ANIs) were calculated among the 50 SAGs, including 21 ASVs, and the ANIs were below 90% among the different ASVs, suggesting that each Methanothrix archaeal strain is a distinct species (Figure 4B). We observed that at least 24 species determined at the threshold of ANI 95% and some species SAG, especially ASV 314, 1,493, and 6,211, were sampled multiple time.

We also observed that two Methanothrix SAGs corresponding to ASV314 were significantly abundant in chemically fertilized paddies. To understand the genomic characteristics of the Methanothrix strains abundant in chemically fertilized paddy fields, we performed a comparative genomic analysis. We identified 10,491 orthologs using a genomic data set of 50 Methanothrix SAGs. Evaluation of the orthologs shared among ASVs confirmed that very few orthologous genes are possessed only by Methanothrix SAGs abundant in chemically fertilized paddy fields, and that genome-wide metabolic functions are highly conserved in all Methanothrix SAGs (Figure 4C). In particular, functional genes directly related to methanogenesis, such as *mcrA*, *fwdB*, *mtbA*, and *mtbC*, were detected in all Methanothrix strains, regardless of their suitability for the fertilization conditions, and no apparent differences in methanogenic potential were expected. Although linear discriminant analysis effect size (LefSe) detected no significant differences in the archaeal abundance, the SAGs corresponding to ASVs (ASV1218, ASV2493, and ASV4356) abundant in organic fertilizer paddies showed relatively high ANIs with ASV314 SAGs abundant in chemical fertilizer paddies, suggesting that although the dominant methanogenic archaeal strains differed depending on fertilizer conditions, the methanogenic archaeal community functionality was retained among the conditions.

## Conclusion

In this study, we successfully developed a pipeline to analyze the genomic diversity of the rice rhizosphere microbiome at single-cell resolution. We obtained 3,237 SAGs in a single experiment, and classified into high-quality (181 SAGs), medium-quality (1,694 SAGs), or low-quality genomes (1,362 SAGs). Comparative genomics using the genomic dataset revealed certain aspects of the microbial community in the paddy ecosystem and will be a valuable resource to understand the principles underlying the rhizosphere ecology in rice and interactions between plants and PGPMs.

## Materials and methods

### Rice cultivation

Field experiments were performed at Shugakuin Imperial Villa in Kyoto city (35°03′13.8′′N 135°48′01.3′′E). The average annual temperature and precipitation in 2021 were 16.9°C and 2034 ml, respectively (Japan Meteorological Agency). Transplantation culture of *Oryza sativa* cultivars Kinuhikari and Nikomaru was performed in loam soil, with a spacing of 20 cm × 15 cm. In plots 1 and 2, methane-fermentation-digested sludge of manure from dairy cows (Yagi Bio-Ecology Center, Kyoto, Japan) was used as basal fertilizer on May 20. The methane-fermentation-digested sludge was obtained with a hydraulic retention time of 50 days at 55°C. In plots 3 and 4, chemical fertilizer 14-14-14 (N 14%, P 14%, K 14%; Japan Agriculture Cooperatives, Kyoto, Japan) was used as basal fertilizer on May 20. In all plots, urea (Japan Agriculture Cooperatives, Kyoto, Japan) was used as ear fertilizer at 2 kg/10 are on August 1. Kusatori Ace L Jumbo, a mixture of cafenstrole, dymron, and bensulfuron-methyl (Mitsui Chemicals Agro Inc., Tokyo, Japan), was used as a pesticide according to the manufacturer’s protocol.

### Sample collection

Soil samples were collected according to a previously described method with slight modifications (Simmons et al., 2018). In brief, rice plants were collected from each plot. The soil loosely attached to the roots was collected as bulk soil. Bulk soil was removed from the roots until there was ~2 mm of soil adhering to the roots, which was defined as the rhizosphere. The roots were sonicated in a buffer (6.75 g KH_2_PO_4_, 8.75 g K_2_HPO_4_, and 1 ml Triton X-100 in 1 L sterile water) with pulses of 160 W for 30 s using BIORUPTOR UCD-250 (Sonicbio Co., Ltd., Kanagawa, Japan). The roots were removed by using sterile tweezers, and then rhizosphere fractions were collected by centrifugation at 4,000 *g* and 4°C for 10 min. The soil samples were flash-frozen until use.

### Soil chemical properties

In each plot, 2 kg of soil samples were collected at three locations and pooled together to reduce variability. The soil samples were desiccated and then filtered using a 2 mm mesh soil sieve. Analysis of soil chemical properties was outsourced to Panasonic Corporation (Osaka, Japan)^1^ (Supplementary Table S1).

### 16S rRNA analysis of soil samples

Bacterial DNA was extracted using an Extrap Soil DNA Kit Plus ver. 2 (BioDynamics Laboratory Inc., Tokyo, Japan). The V3–V4 region of 16S rRNA was amplified using KOD One^®^ PCR Master Mix-Blue- (TOYOBO CO., LTD., Osaka, Japan) and primers 341F (*ACACTCTTTCCCTACACGACGCTCTTCCGATCT*NNNNNCCTACGGGNGGCWGCAG; italic letters indicate adapter sequences) and 805R (*GTGACTGGAGTTCAGACGTGTGCTCTTCCGATCT*NNNNNGACTACHVGGGTATCTAATCC; italic letters indicate adapter sequences; Herlemann et al., 2011). The PCR conditions were as follows: initial denaturation at 94°C for 2 min; 30 cycles of denaturation at 94°C for 10 s, annealing at 65°C for 10 s, and elongation at 72°C for 1 s. In the initial 10 cycles, the annealing temperature decrement per cycle was set to at 1°C. The final cycle was followed by an extension at 72°C for 5 min. The amplified fragments were sent to Bioengineering Lab. Co., Ltd. (Kanagawa, Japan). The PCR products were purified using AMPure XP (Beckman Coulter Inc., CA, United States) and used as a template for second PCR. The second PCR was performed using ExTaq HS (Takara BIO Inc., Shiga, Japan) and primers 2ndF (AATGATACGGCGACCACCGAGATCTACAC-XXXXXXXX-ACACTCTTTCCCTACACGACGC; the Xs indicate index sequences) and 2ndR (CAAGCAGAAGACGGCATACGAGAT-XXXXXXXX-GTGACTGGAGTTCAGACGTGTG; the Xs indicate index sequences). The PCR conditions were as follows: initial denaturation at 94°C for 2 min; 10 cycles of denaturation at 94°C for 30 s, annealing at 60°C for 30 s, and elongation at 72°C for 30 s. The final cycle was followed by an extension at 72°C for 5 min. The resultant products were analyzed using the MiSeq Reagent Kit v 3 (2 × 300 bp; Illumina Inc., CA, United States).

Reads containing the primer sequences were extracted using fastx_barcode_splitter from the FASTX-Toolkit (ver. 0.0.14). Then, the primer sequences were trimmed using fastx_trimer from the FASTX-Toolkit. Bases below Q20 were trimmed, and then reads under 130 bases in length were removed. Denoising, removal of chimeric sequences, and production of amplicon sequence variant (ASV) tables were performed using the dada2 plugin in QIIME 2 (ver. 2021.8; Estaki et al., 2020). Alpha and beta diversity were analyzed and rarefaction curves were generated using the diversity plugin of QIIME 2 with default parameters. A rooted phylogenetic tree for weighted UniFrac was generated using QIIME 2 with default parameters.

### 16S rRNA analysis of methane-fermentation-digested sludge

Methane-fermentation-digested sludge produced in May 2021, September 2021, and March 2022 (Yagi Bio-Ecology Center) was centrifuged at 10,000 *g* and 4°C for 10 min. The resultant pellets were flash frozen and sent to Bioengineering Lab. Co., Ltd. The V3–V4 region of 16S rRNA was amplified using ExTaq HS (Takara BIO Inc.) and the primers as described above. The PCR conditions were as follows: initial denaturation at 94°C for 2 min; 20 cycles of denaturation at 94°C for 30 s, annealing at 55°C for 30 s, and elongation at 72°C for 30 s. The final cycle was followed by an extension at 72°C for 5 min. The PCR products were processed as described above.

### Massively parallel single-cell genome sequencing

SAGs of soil microbes were obtained using the SAG-gel method (Chijiiwa et al., 2020; Arikawa et al., 2021; Nishikawa et al., 2022). Frozen soil was suspended in Dulbecco’s phosphate-buffered saline (DPBS; Thermo Fisher Scientific, MA, United States), and microbial fractions were obtained by density gradient centrifugation using Nycodenz (Serumwerk Bernburg AG, Bernburg, Germany). The concentration of cells was determined using LIVE/DEAD BacLight bacterial viability assay (Thermo Fisher Scientific), and the cells were then suspended in DPBS with 1.5% low-gelling-temperature agarose (Sigma-Aldrich, MO, United States) at 1 cell/capsule. After microfluidic single-cell encapsulation in the capsules, the single-cell-encapsulating gel capsules were recovered in the aqueous phase. Then, gel capsules were immersed in Buffer D2 to denature DNA, and multiple displacement amplification (MDA) was performed for 2 h using the REPLI-g Single Cell Kit (QIAGEN, Hilden, Germany). After MDA, gel capsules were stained with SYBR Green (Thermo Fisher Scientific). FACSMelody cell sorter (Becton, Dickinson and Company, NJ, United States) equipped with a 488 nm excitation laser was used to sort the gel capsules with confirmed DNA amplification into 384-well plates at 1 bead/well. Following capsule sorting, the 384-well plates were stored at −30°C.

For the sequencing analysis, SAG libraries were prepared from the capsule-sorted plates using the QIAseq FX DNA Library Kit (QIAGEN). Ligation adaptors were modified to TruSeq-Compatible Full-length Adapters UDI (Integrated DNA Technologies, Inc., IA, United States). Each SAG library was sequenced using the DNBSEQ-G400 2 × 150 bp configuration (MGITech CO., Ltd., Beijing, China) with the MGIEasy Universal Library Conversion Kit.

### Recovery and assessment of bacterial and archaeal SAGs

For each sequence read obtained from each single cell, low-quality reads were removed using bbduk.sh 38.90 (options: qtrim = r trimq = 10 minlength = 40 maxns = 1 minavgquality = 15 tbo tpe; Bushnell et al., 2017). SAG assembly was then performed with SPAdes 3.15.2 (options: -careful; Bankevich et al., 2012) using the cleaned reads. The obtained SAGs with contigs ≥200 bp in length were evaluated for completeness and redundancy using CheckM 1.1.3 taxonomy_wf (Parks et al., 2015) and for CDS, rRNA, and tRNA detection by Prokka 1.14.6 (default option; Seemann, 2014). The acquired SAGs were classified on the basis of quality according to MISAG standards (Bowers et al., 2017). All SAGs were assessed using QUAST 5.0.2 (default options; Gurevich et al., 2013), and their taxonomies were classified using GTDB-Tk 1.4.1 classify_wf (Chaumeil et al., 2019). Phylogenetic trees obtained using GTDB-Tk infer were visualized by iTOL 6.5.7 (Letunic and Bork, 2021). By performing a BLAST search (blastn 2.9.0+) of ASV sequences obtained by QIIME2 and 16S rRNA gene sequences detected in SAGs by Prokka, SAGs were matched to ASVs in the 16S rRNA analysis.

### Genome analysis of archaea belonging to the genus Methanothrix

Comparative genomic analysis was performed on the acquired high-quality (HQ) and medium-quality (MQ) Methanothrix SAGs. First, 50 SAGs with 16S rRNA sequences showing ≥97% homology to Methanothrix ASVs were identified, and the average nucleotide identity (ANI) between Methanothrix SAGs was calculated using FastANI (Jain et al., 2018). The abundances of each Methanorix organism were estimated from 16S rRNA analysis data, and differential abundance analysis was conducted by LefSe with default parameters (Segata et al., 2011). Ortholog analysis of Methanothrix SAGs was then performed using Orthofinder 2.5.2 (default options; Emms and Kelly, 2019). The orthologous genes that were significantly more abundant in the Methanothrix SAGs obtained from specific conditions were detected by the fisher exact test and analyzed by InterProScan 5.54 (options: -appl Pfam; Jones et al., 2014) and KofamScan 1.3.0 (default option; Aramaki et al., 2020) for functional annotation.

## Data availability statement

The datasets presented in this study can be found in online repositories. The names of the repository/repositories and accession number(s) can be found at: https://www.ncbi.nlm.nih.gov/, PRJNA864623; https://www.ncbi.nlm.nih.gov/, PRJNA869948.

## Author contributions

WA, SM, MH, HT, TM, and MU conceived the project. SM, KM, SH, and TM contributed to the field experiment and sample collection. WA, SM, MK, YN, MH, and HT contributed to NGS analysis. All authors contributed to the article and approved the submitted version.

## Funding

This research was supported by JST COI-NEXT (grant number JPMJPF2008), Japan.

## Conflict of interest

Kyoto Agriculture Research Institute KARI provided support in the form of salaries for KM, SH, and TM. MH and HT are shareholders in bitBiome, Inc., which provides single-cell genomics services using the SAG-gel workflow as bit-MAP. MK is employed at bitBiome, Inc. SM is employed at WORLD INTEC CO., LTD.

The remaining authors declare that the research was conducted in the absence of any commercial or financial relationships that could be construed as a potential conflict of interest.

## Publisher’s note

All claims expressed in this article are solely those of the authors and do not necessarily represent those of their affiliated organizations, or those of the publisher, the editors and the reviewers. Any product that may be evaluated in this article, or claim that may be made by its manufacturer, is not guaranteed or endorsed by the publisher.

## Footnotes

^1^ https://news.panasonic.com/jp/press/data/2020/05/jn200528-3/jn200528-3.html
